# You Are Where You Live: The Interrelationship of Air Pollution, Address, and Walkability

**DOI:** 10.1289/ehp.117-a505b

**Published:** 2009-11

**Authors:** Tanya Tillett

**Affiliations:** **Tanya Tillett**, MA, of Durham, North Carolina, is a writer/editor for *EHP*. She has been with *EHP* since 2000 and has represented the journal at national and international conferences

Exposure to air pollution adversely affects human health by triggering or exacerbating a number of conditions such as asthma and heart disease. Likewise, physical inactivity has been linked to negative health consequences including heart disease and diabetes. Now for the first time a new study offers a quantitative analysis of the intersection between neighborhood “walkability”—or how conducive the neighborhood is to walking—and exposure to air pollutants **[*EHP*** **117:1752–1759; Marshall et al.]**.

The authors analyzed concentration estimates of nitric oxide (NO), a marker of fresh vehicle exhaust, and ozone (O_3_), a secondary pollutant formed in the atmosphere from vehicle emissions and other pollutants. Concentrations were estimated for the months of May–September. The authors then compared those levels against neighborhood walkability scores, which they calculated for 89% of the postal codes in Vancouver, British Columbia (the average postal code for the city comprises 39 people or 0.05 km^2^). Walkability scores were calculated based on residential density, intersection density, retail floor-area, and land-use mix of the postal code area. The study did not measure people’s daily exercise levels or their exposure to air pollution—both of which may vary within a neighborhood and even within a single household.

The authors report that lower-income areas tended to have higher walkability scores and lower O_3_ concentrations than did higher-income areas, but had higher NO concentrations. That finding reflects the tendency of lower-income areas to fall in busier urban areas whereas middle-income areas tend to fall farther from the city center. “Sweet spot” neighborhoods with high walkability and low pollution tended to be located near but not at the city center. They typically featured highly connected streets, mixed land uses, sidewalks, and an absence of large parking lots; they also tended to be in higher-income areas. “Sour spot” neighborhoods with high pollution and low walkability tended to be located far from the city center.

The findings indicate that walkable urban settings can offer health benefits but may also come with health costs when exposure to air pollution is considered. The authors write that high NO exposure may occur in low-income areas and in areas where walking, biking, and other forms of “active transportation” are encouraged, and that strategies are required to mitigate exposure to high concentrations of air pollutants. This type of analysis could be used to monitor changes over time in future urban development and redevelopment projects.

## Figures and Tables

**Figure:**
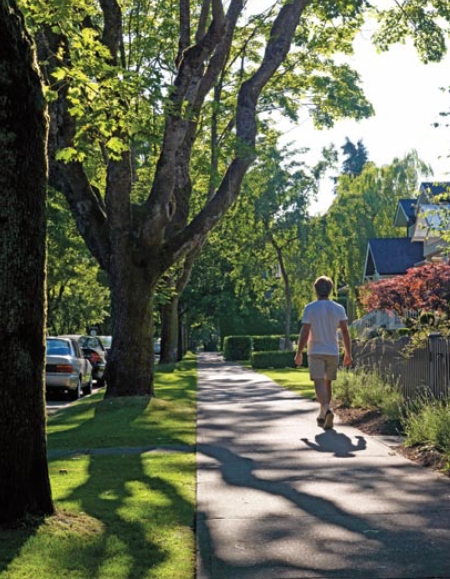
Vancouver, British Columbia

